# Facilitating L2 writers’ metacognitive strategy use in argumentative writing using a process-genre approach

**DOI:** 10.3389/fpsyg.2022.1036831

**Published:** 2022-11-15

**Authors:** Yu Huang, Lawrence Jun Zhang

**Affiliations:** ^1^School of Foreign Languages and Literature, Wuhan University, Wuhan, China; ^2^Faculty of Education and Social Work, University of Auckland, Auckland, New Zealand

**Keywords:** metacognitive strategy use, L2 writing, process-genre appraoch, argumentative writing, think-aloud protocols

## Abstract

This paper reports on an empirical study that examined changes in L2 writers’ perceived use of metacognitive strategies after receiving a process-genre writing instruction. Following a mixed-methods approach, this study was conducted in two intact College English classes at a university in China. Participants were 72 first-year undergraduates, with an experimental group (*n* = 40) taught by the process-genre writing approach and a comparison group (*n* = 32) receiving conventional writing instruction. A Likert-scale questionnaire was used to examine students’ changes in their conceptualized metacognitive strategies. Think-aloud protocols were conducted to gain an in-depth understanding of students’ application of metacognitive strategies and genre knowledge in performing writing tasks. Findings revealed that the process-genre instruction had a significantly positive impact on the “considering the audience” factor, and students’ conception of the audience was clearer and more diversified. An in-depth analysis of the think-aloud protocols showed that the participants incorporated the acquired metacognitive strategies and genre knowledge in completing writing tasks, with more pre-task planning time focused on both global and local aspects. Students’ metacognitive monitoring also shifted from surface-level lexical and grammar regulation to discourse-level text control.

## Introduction

It is widely acknowledged that writing is a complex and multidimensional sociocognitive activity in which writers need to deal with linguistic, contextual and content knowledge, as well as the metacognitive control of the cognitive processes (Bereiter and Scardamalia, 1987; Hyland, 2003; Graham and Perin, 2007; Hayes, 2012; Zhang, 2013, 2021). Writing is even more challenging for EFL learners than English natives as the former has little sociocultural exposure to the authentic English language and different types of genres. Many scholars have underscored the importance of developing L2 writers’ genre awareness through consciousness-raising, context-building, modeling and explicit analysis of rhetorical structures (Swales, 1990; Negretti and Kuteeva, 2011; Yeh, 2015; Negretti and McGrath, 2018). Skilled writers actively deploy and regulate strategies to search for information, plan for content and rhetorical structures and make appropriate lexical-grammatical choices in consideration of their audience and communicative purposes throughout the writing process (Raphael et al., 1989; Mitchell and Pessoa, 2017; Han and Hiver, 2018; Teng and Zhang, 2020). Novice writers, however, are more concerned about knowledge telling (Bereiter and Scardamalia, 1987), grammar correctness and linguistic complexity (Kellogg, 2008). Even though some students learned genre knowledge and strategies, they tend not to use them for performing tasks (Lee and Mak, 2018). To this end, it is essential to facilitate writers’ use of metacognitive strategies in L2 writing instruction so that writers can actively monitor and regulate the various aspects of writing during the recursive process of planning, drafting, revising and editing, thus ultimately improving their writing proficiency (De Silva and Graham, 2015; Zhang and Zhang, 2018; Qin and Zhang, 2019; Chen et al., 2022; Teng et al., 2022).

Predictive effects of self-regulated strategy use on L2 writers’ writing proficiency have highlighted the critical role of metacognition in language learning (Teng and Zhang, 2016; Teng and Huang, 2019), and it is even more so when it comes to genre-based writing instructions (Yeh, 2015; Kessler, 2021; Sun and Zhang, 2022a,b). It has been argued that metacognitive instruction should be deliberately taught in the genre classroom (Negretti and McGrath, 2018; Teng, 2022). The process-genre approach, with its advantage of integrating genre and explicit strategy instruction throughout the recursive processes, draws much attention from L2 writing researchers (Badger and White, 2000; Huang and Zhang, 2020; Rahimi and Zhang, 2021; Wang and Huang, 2022). The process-genre approach includes many features common to other strategy-based instructions. It stresses the explicit instruction of strategies for planning, drafting, editing and revising, including (1) engaging students to brainstorm ideas and outline their essay structures in prewriting activities; (2) modelling and drafting collaboratively with students to develop their ability to synthesize information and making lexical, grammatical and structural choices; (3) involving students in the editing and revising processes with reference to the argumentative writing checklist. Previous studies have suggested that the process-genre approach is an effective and enabling approach to improving L2 writers’ genre awareness (Deng et al., 2014; Han and Hiver, 2018), writing engagement (Rahimi and Zhang, 2021), motivational beliefs (Teng, 2022) and writing quality (Huang and Zhang, 2020). Nevertheless, little has been known about its impact on writers’ metacognitive strategy use. To fill this gap, the present study investigates how students’ metacognitive strategies can be facilitated after receiving the process-genre writing instruction.

## Literature review

### Metacognition and genre awareness

Metacognition has not been explicitly addressed in most genre-based pedagogies. Alexander and Murphy (1999) criticized genre-based writing instruction for focusing merely on the macrostructure analysis or linguistic features without activating students’ metacognition to apply genre knowledge in writing practices. Similarly, other researchers (e.g., Schraw, 2002; Yeh, 2015; Lee and Mak, 2018) argued that explicit instruction in genre knowledge alone is inadequate to help students write effectively. Without metacognitive regulation/strategies, students often have difficulty applying genre knowledge when performing the task. Additionally, Hyland (2007) pointed out that the genre-based approach should not be a top-down approach in which teachers merely model sample texts and give explicit instruction about genre knowledge. It should also include the development of students’ metacognitive skills so that students are encouraged to thoughtfully and critically engage in the writing processes from writers’ perspectives.

In recent years, a growing number of researchers have perceived metacognition as part of learners/writers’ genre awareness development (e.g., Negretti and Kuteeva, 2011; Tardy, 2016; Negretti and McGrath, 2018; Kessler, 2021; Zhang and Zhang, 2021). Negretti and Kuteeva (2011) investigated the metacognitive genre awareness among a group of pre-service English teachers using an ESP genre-based academic reading and writing instruction. Results suggested that all participants developed declarative and procedural metacognitive knowledge, but only a few demonstrated conditional knowledge of the genre in their reading and writing tasks. Similarly, in a longitudinal case study, Kessler (2021) investigated changes in six focal students’ metacognitive genre awareness on writing the office memo and found that students’ metacognitive genre awareness was developed multidimensionally but unevenly. Participants produced more comments about metacognitive knowledge than how they regulate their writing processes.

Research also suggested that students’ metacognitive genre awareness can be facilitated through metacognitive tasks. Yeh (2015) incorporated metacognitive genre-based tasks into an online writing system to support students going through the planning, monitoring, evaluating and revising stages. The study investigated how such a system can foster the application of genre knowledge in graduate students’ academic writing. Through analyzing different versions of students’ research proposals, online actions logs, discussion transcripts and open-ended questionnaires, it was found that students’ academic writing quality improved after experiencing the metacognitive processes in the program. Particularly, the collaborative efforts throughout the metacognitive processes supported students’ understanding of the academic genre, identification of the problems of unstructured academic papers and application of their genre knowledge to academic writing. More recently, Negretti and McGrath (2018) investigated how genre knowledge and metacognition can be scaffolded in a genre-based course with two metacognitive tasks. The two doctoral students were asked to describe the writing contexts and genres at the beginning of the course and draw a visual concept of the research genre in their specific science community at the end of the course. Results suggested that the metacognitive tasks can encourage students to integrate various aspects of their genre knowledge, consider reader expectations, contexts, and conventions, and make strategic choices.

These studies suggested the effectiveness of metacognition in developing students’ learning to write ability in a specific genre in terms of improving their writing quality and metacognitive genre awareness. Nevertheless, not all facets of metacognition developed evenly among learners. It is also noted that conditional knowledge, more closely tied to aspects of metacognitive regulation, was less facilitated. Thus, it is critical for scholars to look into how students’ metacognitive regulations can be developed in different instructional contexts.

### Metacognition and process-genre instructional framework

The process-genre approach is an eclectic approach that incorporates the strengths of both the process and genre-based approaches (Badger and White, 2000). Instead of viewing writing as a linear and simple activity, the process-genre teaching model emphasizes the cognitive, linguistic, affective and sociocultural nature. A salient advantage of the process-genre approach is that it draws on the explicit and consciousness-raising nature of the genre-based approach while incorporating strategy instruction into the writing processes (Huang and Zhang, 2020; Teng, 2022).

**Figure 1:**
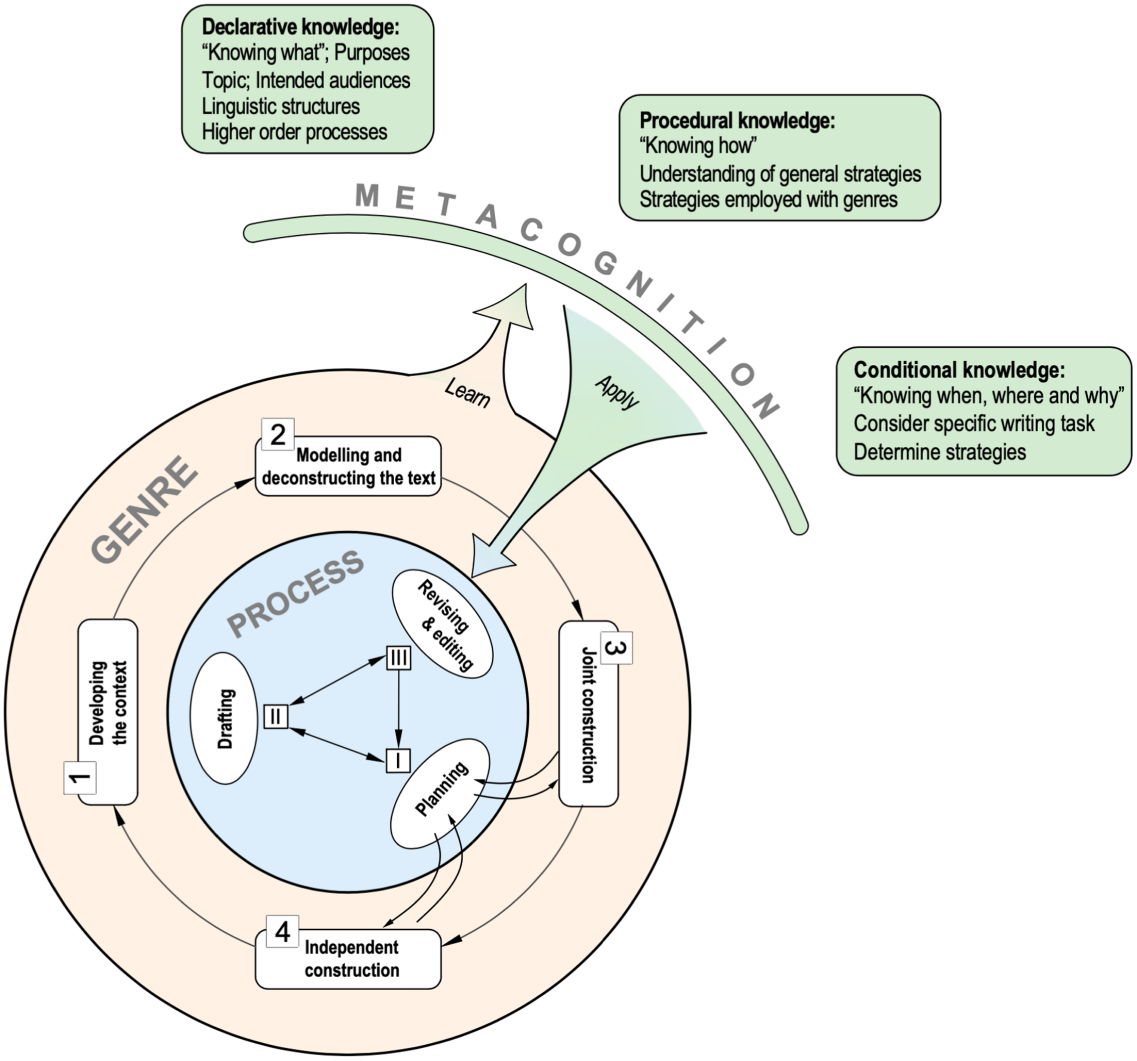
Schematic illustration of the process-genre instructional framework (Adapted with permission from Huang and Zhang, 2020).

The Systemic Functional Linguistics (SFL)-informed process-genre instructional framework incorporates the stages of the Teaching and Learning Cycle in which students are scaffolded in the manageable recursive processes of writing. Figure 1 is an adaption of the process-genre instructional framework of Huang and Zhang (2020), with an illustration of how metacognition can be incorporated into the pedagogical process of writing instruction (Harris et al., 2010). The teacher started the class by building the writing context, helping students understand the purposes of writing and the intended audiences. After building learners’ schema of the specific genre, the teacher moves on to the stage of modelling and deconstruction, where language features and rhetorical structure of the particular genre are introduced to the students through teacher-guide and students-led model essay analysis. These stages enhance students’ declarative knowledge (e.g., audience, purposes, rhetorical structures, and language choices) to complete the task. During the joint construction stage, the instruction moves to the process level, where students’ procedural knowledge about writing is developed. Through modelling what expert writers do during the writing process, the teacher explicitly teaches students strategies for efficacious planning (e.g., goal setting, idea generating and idea structuring), text production (e.g., linguistic and content input), editing and revising (Harris et al., 2010; Lam, 2014). Strategies commonly employed in the argumentative genre are also highlighted (e.g., supporting argument with specific and reliable evidence, utilizing effective refutation, identifying fallacies, etc.). In the independent construction stage, students are given related topics in the same genre. Independent writing is expected to enhance students’ procedural knowledge, enabling the writer to consider the writing task and determine the employment of different strategies to achieve their writing goals.

Therefore, it is hypothesised that the process-genre approach, juxtaposing the development of genre awareness and explicit strategy instruction, can further increase students’ metacognitive knowledge and regulation (Lam, 2014). Given the significant roles of process-genre writing instruction in developing writers’ metacognition, further research is needed to investigate whether students’ metacognitive strategy use can be facilitated as a result of the process-genre writing instruction and how students use their metacognitive strategies to apply their genre awareness during the writing process. Such findings may offer pedagogical insights into strategy instruction in genre learning in L2 contexts. Therefore, the present study aims to address two research questions.

RQ1 What is the impact of the process-genre writing intervention on L2 writers' perceived metacognitive strategy use?

RQ2 To what extent do L2 learners deploy their metacognitive strategies while performing the writing task?

## Materials and methods

This classroom-based intervention research followed a mixed-methods approach. Quantitative data were collected from the Metacognitive Strategy Questionnaires in the pre-, post-, and delayed post-tests; qualitative data were drawn from the think-aloud protocols before and after the intervention.

### Participants

The study was conducted in two intact College English classes at a comprehensive university in Central China. 72 students in both classes agree to participate in the study. The two classes were randomly assigned into the experimental group (*n* = 40) and control group (*n* = 32), with the former taught by the process-genre writing approach and the latter receiving conventional writing instruction. Participants were from intermediate-level classes, and their English proficiency level was equivalent to Common European Framework of Reference (CEFR) B2,^1^ assessed by the English Placement Test. At the time of the study, they had been learning the English language for 10 years on average (EG: *M* = 10.1, *SD* = 2.16; CG, *M* = 10.4, *SD* = 1.7). Their prior L2 writing learning experience is limited, and they receive mainly exam-oriented writing instructions. The participants were the same as Huang and Zhang (2020), but different datasets were used in the present study. In this study, questionnaire data were collected from 72 students in both groups. Two participants from the experimental group were recruited using purposive sampling for think-aloud protocols. The selection of think-aloud participants was based on three criteria: (1) average-achieving students, (2) demonstrate the ability to perform think-aloud protocols, and (3) have sufficient time to attend the think-aloud protocols. All participants were involved voluntarily.

### Measures

#### L2 writing metacognitive strategy questionnaire

The present study used the Metacognitive Strategy Questionnaire (MSQ) in the pre-, post-and delayed post-tests to examine changes in students’ use of metacognitive strategies in the writing process. The L2 writing MSQ was adopted and modified from Amani (2014) for two reasons. First, unlike other general measures of metacognitive strategy questionnaires, the development of MSQ is based precisely on the L2 writing context for the argumentative task, which can fit into our research aims and contexts. Second, Amani (2014) undertook a validation process using qualitative and quantitative methods to develop L2 writing MSQ, which can guarantee its validity and reliability. The L2 writing MSQ contains 21 items, measuring four types of metacognitive strategies: planning, considering the audience, monitoring and evaluating (see Appendix A), based on Flavell et al.’s (2002) taxonomy.

The researcher translated the questionnaire items into Chinese as it was considered that, as students were first-year university students, they might have difficulty understanding the meaning of the items in English. The original English version of the MSQ and its Chinese version was sent to the first author’s Chinese colleagues to guarantee accuracy and intelligibility. The Chinese MSQ was also piloted on six non-participant first-year students enrolled in the same course. They were invited to complete the questionnaire while thinking aloud. Any difficulty in understanding or ambiguity was noted on the item, and the researcher was asked for elaboration. The validation process resulted in a minor revision of the wording of the MSQ. After modification, the MSQ was piloted with 35 students for reliability analysis using the Cronbach’s alpha coefficient. The Cronbach’s alpha values for planning, considering the audience, monitoring and evaluating are α = 0.801, 0.813, 0.821, and.808, respectively.

#### Think-aloud protocols

The think-aloud protocol is an introspective research method in which participants verbalize whatever comes into their minds while performing a task (Ericsson and Simon, 1993). Researchers often audio-and video-record the think-aloud process so that they can replay and refer to what participants have verbalized (Gu et al., 2005; Yang et al., 2014). Think-aloud protocol method has been widely used in writing research in L1 and L2 contexts (Flower and Hayes, 1981; Qi and Lapkin, 2001; Manchón, 2009; Kim and Bowles, 2019; Suh, 2020), as it explicitly reveals writers’ detailed cognitive process while performing the tasks (Leighton and Gierl, 2007). Despite concerns about the reactivity and veridicality of this method (Sachs and Polio, 2007; Yang et al., 2014), many researchers view it as a trustworthy methodological choice in second-language research if it is operated and interpreted with care (Bowles, 2010; Zhang and Zhang, 2020).

In this study, concurrent think-aloud protocols were used over other retrospective research methods because they are direct in revealing in-depth how participants employ their knowledge and use of metacognitive strategies in the ongoing writing process. The qualitative data produced in think-aloud sessions were triangulated with the quantitative data from the questionnaires.

### Treatment

Writing instruction was provided as part of a four-semester College English course, a compulsory course taken consecutively by undergraduate students during the first 2 years of their four-year undergraduate program. The present study was conducted in a regular classroom during the second semester. A total of 26 90-min sessions were conducted over 17 weeks, among which six sessions were allotted to L2 writing instruction. Argumentative writing was chosen as the target genre for both groups based on an analysis of the curriculum and students’ needs. Topics for argumentative writing instruction were drawn from three textbook themes: (1) technology and education, (2) career choices and (3) animal protection. Both groups received instruction on the same topics and an equal amount of time for instruction. The only difference was the way writing instruction was carried out. The process-genre instruction in this study was designed following the stages of the process-genre teaching model, as reported in Huang and Zhang (2020), where both conceptual and strategic content was taught. Students learned genre knowledge about the writing context, purposes and audiences, argumentation structures, the logical flow of the text and research skills to collect information from reliable sources. Strategic content includes collaborative writing, peer review and revision skills. For the comparison group, writing instruction followed the commonly practised product-oriented way that directs students’ attention to language knowledge and model essay imitation. Students are required to submit their essays to an online evaluation platform, with the teacher being the only reader.

### Data collection

Participants from both groups completed the L2 writing MSQ before, immediately after and 4 weeks after the instruction.

Two students from the experimental group were invited to participate in the case study in which three think-aloud protocols were collected at each test while performing the argumentative writing task. Participants’ verbalization during the think-aloud protocols was audio-recorded with their permission. As the think-aloud sessions needed to be done in the laboratory booths, they were conducted 1 day before each formal test. The two participants did the task simultaneously in different booths because it reduced their psychological pressure. Before the think-aloud sessions, the researcher conducted a training session for the participants on how to verbalise their thinking process. The reason why such a technique was used as part of the informant training was that prior studies have successfully used it even with primary schoolchildren (e.g., Gu et al., 2005; Zhang et al., 2008). A sample topic that is similar to the test task was given to the participants to practice. When participants demonstrated their mastery of verbalising their thoughts, the formal think-aloud session ensued. The participants were told to articulate their thoughts in either Chinese or English, or a mixture of both, and not to worry about the correctness of the utterance while performing the task. There was no time limit for the writing task to reduce the participants’ anxiety (Table 1).

**Table 1:** Data collection procedure.

Week of semester	Phase	Data collected	Participants
Week 1–3	Pilot study	Background questionnaire	*n* = 72
		Metacognitive strategy questionnaire	*n* = 35
Week 4	Pre-test	Think-aloud protocols	EG (n = 2)
		Metacognitive strategy questionnaire	EG (n = 40) CG (n = 32)
Week 4–9	Intervention	Six writing instruction sessions	EG (n = 40) CG (n = 32)
Week 10	Post-test	Think-aloud protocols	EG (n = 2)
		Metacognitive strategy questionnaire	EG (n = 40) CG (n = 32)
Week 16	Delayed post-test	Metacognitive strategy questionnaire	EG (n = 40) CG (n = 32)

### Data analysis

#### L2 writing metacognitive strategies questionnaire

The MSQ consists of four factors: planning, considering the audience, monitoring and evaluation. Participants’ choices on the 7-point scale were coded as scores for each item. The mean score was used as an indicator of participants’ metacognitive strategies. To compare students’ responses to the four factors, items within each factor were averaged. These averaged values were subjected to Mixed ANOVA analysis, followed by Simple Effect Analysis. The within-subject comparison was applied to time (pre-, post-, and delayed post-test) and between-subject comparison to instructional conditions (treatment and comparison). However, the data for some variables of the comparison group do not meet the assumption of normality, mixed ANOVA could not be conducted. Instead, one-way repeated measures ANOVA or Friedman test was used to determine the changes in metacognitive strategies over time within each group. Further follow-up Paired samples t-tests or Wilcoxon Signed Ranks tests were used to examine the pairwise comparisons within each group. Independent Samples t-tests or Mann–Whitney *U* tests were run to explore the differences between groups across three tests further.

#### Think-aloud protocols

The think-aloud data were analyzed qualitatively following deductive thematic analysis (Braun and Clarke, 2011), reporting how each individual participant employed the metacognitive strategies and his/her acquired genre knowledge in the actual writing process over time. The audio-taped data, collected at the pre-and post-tests, were transcribed by the researcher and crosschecked by a research assistant. A coding scheme was developed based on Flavell et al.’s (2002) model of metacognition and SFL-informed genre theory (Halliday and Hasan, 1989; Rose and Martin, 2012). To guarantee the reliability of the coding, the researcher independently coded two protocols with a research assistant, as a trial run. Where there was disagreement, a face-to-face discussion was carried out until an agreement was reached.

## Findings

This section first provides findings from the Metacognitive Strategy Questionnaire (RQ1), followed by an in-depth analysis of think-aloud protocols from two participants of the experimental group, showing individuals’ deployment of their metacognitive strategy use during the writing process (RQ2).

### Findings from MSQ

**Figure 2:**
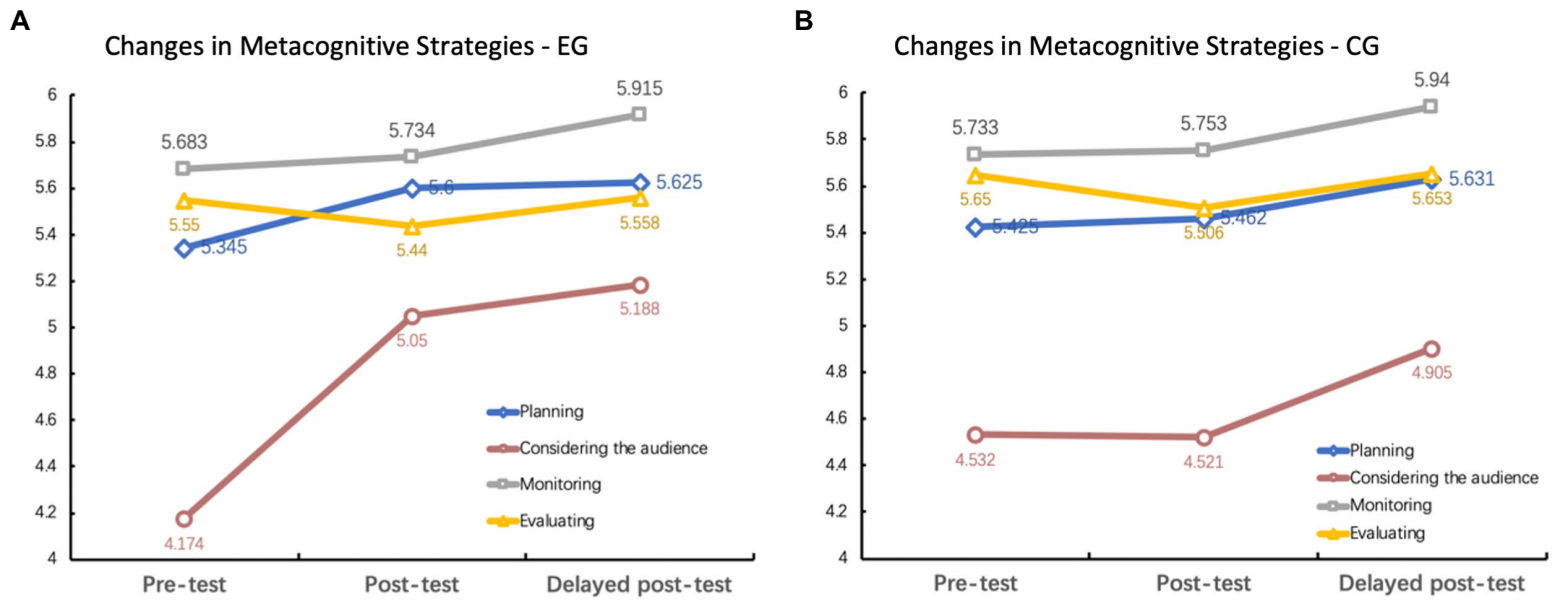
Changes in metacognitive strategy use: **(A)** experimental group; **(B)** comparison group.

Descriptive statistics of learners’ reported metacognitive strategy use between two conditions in the pre-, post-and delayed post-tests are shown in Table 2 and Figure 2.

**Table 2:** Descriptive statistics for metacognitive strategies questionnaire.

Variables	Group	*N*	Pre-test		Post-test		Delayed post-test	
			Mean	SD	Mean	SD	Mean	SD
Planning	EG	40	5.345	0.807	5.600	0.873	5.625	0.930
	CG	32	5.425	0.833	5.462	0.879	5.631	0.963
Considering the audience	EG	40	4.174	1.390	5.050	1.018	5.188	1.206
	CG	32	4.532	1.176	4.521	1.287	4.905	1.156
Monitoring	EG	40	5.683	0.670	5.734	0.748	5.915	0.736
	CG	32	5.733	0.797	5.753	0.800	5.940	0.836
Evaluation	EG	40	5.550	0.936	5.440	1.074	5.558	0.888
	CG	32	5.650	0.879	5.506	0.993	5.653	0.857

### Effects on planning

The planning subscale addresses participants’ conscious attention to the preparatory steps before undertaking the task. There are five items in the planning subscale. The average score of the five items was calculated and assigned as the value for the planning subscale. A repeated-measures ANOVA determined that the planning score was not significantly different in the pre-, post-and delayed post-test for the experimental group, *F* (2, 78) = 1.971, *p* = 0.146, with a small effect size, 𝜂_𝜌_^2^ = 0.048. *Post hoc* tests using the Bonferroni correction revealed that the process-genre approach elicited a slight increase in planning from pre-test to post-test (Mean = 5.345, *SD* = 0.807 vs. Mean = 5.600, *SD* = 0.873, respectively), which was not statistically significant (*p* = 0.354). In the delayed post-test, the planning had increased to Mean = 5.625, *SD* = 0.930, but no statistical difference was found with either post-test (*p* = 1.000) or pre-post (*p* = 0.326), implying that the process-genre approach did not help students develop significantly in planning. Neither were the effects on planning identified after 6 weeks.

To examine the changes in planning for the comparison group, the Friedman test was carried out because the data did not meet the normality assumption. Results show that there was not a statistically significant difference in planning for the comparison group over the three tests, χ^2^(2) = 2.835, *p* = 0.242. *Post hoc* analysis with Wilcoxon signed-rank tests was conducted with a Bonferroni correction applied, resulting in a significance level set at *p* < 0.017. The median planning value for the comparison group in pre-, post-and delayed post-test was 5.4 (5 to 6.15), 5.6 (5 to 6) and 5.9 (5.25 to 6.2), respectively. There were no significant differences between the pre-test and post-test (*Z* = −0.183, *p* = 0.855) or between the pre-test and delayed post-test (Z = −1.732, *p* = 0.083). No statistically significant difference was found in the post-test and delayed post-test (*Z* = −1.623, *p* = 0.105).

The between-group comparison was made by using the Mann–Whitney *U* test. Results show that no significant differences were found in “planning” between the IG and CG at the three tests (pre-test, *Z* = −1.349, *p* = 0.177; post-test, *Z* = −0.506, *p* = 0.613; delayed post-test, *Z* = −0.365, *p* = 0.715).

### Effects on considering the audience

The “considering the audience” factor includes three items, which evaluate participants’ awareness of a perceived audience. As the assumption of normality and homogeneity was met, mixed ANOVA was carried out to determine the effect of both the process-genre approach and the conventional approach on considering the audience factor. However, the assumption of sphericity was not met, so the mixed ANOVA was run with a Greenhouse–Geisser correction. Results show that there was a significant main effect of Time on considering the audience factor, *F* (1.792, 140) = 12.365, *p* < 0.001, 𝜂_𝜌_^2^ = 0.150. The effect indicates that if we ignore the difference in groups, considering the audience value in some of the three tests were significantly different from others. However, no significant difference was found in the main effect of Group, *F* (1, 70) = 0.129, *p* = 0.0721, 𝜂_𝜌_^2^ = 0.002, on students considering the audience factor. The effects tell us that if we ignore the testing time variable, students’ considering audience awareness in the EG was not significantly different from those of the comparison group. It shows an interaction effect for Time × Group, *F* (1.792, 140) = 3.217, *p* = 0.049, 𝜂_𝜌_^2^ = 0.044.

The Time × Group interaction effect was further analyzed by using a simple effect analysis. The within-subject pairwise comparison reveals that there was a significant difference in students’ overall writing scores between the pre-test and the post-test (*p* < 0.001), and between the pre-test and the delayed post-test (*p* < 0.001) for the experimental group, but no significant difference was identified between the post-test and the delayed post-test (*p* = 1.000). This indicates that the treatment had a significant effect on the experimental group in the post-test and the effect was retained in the delayed post-test. However, no significant difference was found for the comparison group over the three-time points (pre-*vs* post-test, *p* = 1.000; post-*vs* delayed post-test, *p* = 0.645). This implies that conventional writing instruction had little effect on students’ awareness of considering the audience.

Additionally, the result of the between-subject comparison provides that, at baseline, there was no significant difference between the experimental group and the comparison group on considering the audience factor in the pre-test (*p* = 0.308), post-test (*p* = 0.239) or delayed post-test (*p* = 0.206).

### Effects on monitoring

The monitoring factor elicits students’ perception of their online awareness of comprehension and task performance (Flavell et al., 2002). The average value of the eight items was calculated and compared. To investigate the effect of the process-genre writing instruction on monitoring skills for the experimental group, a repeated-measures ANOVA was conducted. Results indicate that students’ monitoring skill was not significantly different across the three tests, *F* (2, 78) = 3.235, *p* = 0.080, with a small effect size, 𝜂_𝜌_^2^ = 0.077. *Post hoc* tests using the Bonferroni correction revealed that the process-genre approach helped increase students’ awareness of audience from pre-test to post-test (Mean = 5.683, *SD* = 0.670 vs. Mean = 5.734, SD = 0.748, respectively), which was not statistically significant (*p* = 1.000). In, the delayed post-test, the awareness of audience had been increased to Mean = 5.915, *SD* = 0.736, but a statistical difference was not detected compared to either to the pre-test (*p* = 0.239) or the post-test (*p* = 0.537). Therefore, we can conclude that the process-genre approach did not help students significantly develop their metacognitive monitoring, and the effect was not visible after 6 weeks of instruction.

To examine the changes in the monitoring factor for the comparison group, the Friedman test was carried out because the normality assumption was not met. Results show that there was no statistically significant difference in planning for the comparison group over the three tests, χ^2^(2) = 2.581, *p* = 0.275. *Post hoc* analysis with Wilcoxon signed-rank tests was conducted with a Bonferroni correction applied, resulting in a significance level set at *p* < 0.017. The median of the monitoring factor for the comparison group in pre-, post-and delayed post-test were 6.00 (5.125 to 6.250), 5.880 (5.50 to 6.25) and 5.94 (5.75 to 6.5), respectively. There were no significant differences between the pre-test and post-test (*Z* = −0.400, *p* = 0.689) or between the pre-test and delayed post-test (*Z* = −1.820, *p* = 0.069). Also, no statistically significant difference was observed in the post-test and delayed post-test (*Z* = −1.776, *p* = 0.076). The findings indicated that neither the process-genre approach and nor the conventional approach helped students to improve their metacognitive strategies in monitoring.

Between-group comparison using a Mann–Whitney *U* test show that no significant differences were found in “monitoring” between the EG and CG at the three tests (pre-test, *Z* = −0.795, *p* = 0.426; post-test, *Z* = −0.420, *p* = 0.674; delayed post-test, *Z* = −0.324, *p* = 0.746).

### Effects on evaluation

The evaluation factor refers to students appraising the products and regulatory processes of one’s learning. Five items in the questionnaire measured this factor. To investigate the effect of the process-genre writing instruction on students’ evaluation skills for the experimental group, a repeated-measures ANOVA was conducted. Results indicated that students’ evaluating strategy was not significantly different across the three tests, *F* (2, 78) = 0.303, *p* = 0.740, with small effect size, 𝜂_𝜌_^2^ = 0.008. *Post hoc* tests using the Bonferroni correction revealed that the process-genre approach helped increase students’ evaluation from pre-test to post-test (Mean = 5.550, *SD* = 0.936 vs. Mean = 5.440, *SD* = 1.074, respectively), which was not statistically significant (*p* = 1.000). In the delayed post-test, the awareness of the audience had been increased to Mean = 5.558, *SD* = 0.888, but statistical differences were not found compared to either the pre-test (*p* = 1.000) or the post-test (*p* = 1.000). Therefore, it can be concluded that the process-genre approach did not significantly help students develop evaluation skills, and the effect was not visible after 6 weeks of instruction.

The Friedman test was carried out to examine the changes in evaluation for the comparison group because the data did not meet the normality assumption. Results show no statistically significant difference in evaluation for the comparison group over the three tests, χ^2^(2) = 1.398, *p* = 0.497. *Post hoc* analysis with Wilcoxon signed-rank tests was conducted with a Bonferroni correction applied, resulting in a significance level set at *p* < 0.017. The median planning value for the comparison group in pre-, post-and delayed post-test was 5.80 (5.40 to 6.20), 5.7 (4.80 to 6.20) and 5.80 (5.40 to 6.20), respectively. There were no significant differences between the pre-test and post-test (*Z* = −0.796, *p* = 0.426) or between the pre-test and delayed post-test (*Z* = −0.034, *p* = 0.973). Also, no statistically significant difference was found in the post-test and delayed post-test (*Z* = −1.101, *p* = 0.271).

A between-Group comparison was made by using the Mann–Whitney *U* test. Results show that no significant differences were found in “evaluating” between the EG and CG at the three tests (pre-test, *Z* = −0.631, *p* = 0.528; post-test, *Z* = −0.579, *p* = 0.562; delayed post-test, *Z* = −0.723, *p* = 0.470).

Table 3 describes a summary of the findings. It is suggested that the process-genre instruction was found to have a significant impact on participants’ responses to items in considering the audience factor, and the effect was retained after 6 weeks. However, no significant difference was found in the three other factors of planning, monitoring and evaluation. No significant difference was observed among either of the four factors of metacognitive strategy for the comparison group.

**Table 3:** Effects on metacognitive strategy use for the EG and the CG.

	Pre-vs. post-test				Post-vs. delayed post-test				Pre-vs. delayed post-test			
	EG		CG		EG		CG		EG		CG	
	*SE*	*p*	Z	*p*	*SE*	*p*	*Z*	*p*	*SE*	*p*	*Z*	*p*
Planning	0.156	0.354	−0.183	0.855	0.136	1.000	−1.623	0.105	0.170	0.326	−1.732	0.083
Considering the audience	0.193	0.000^**^	−0.926	0.354	0.184	1.000	−1.564	0.118	0.236	0.000^**^	−0.1388	0.165
Monitoring	0.127	1.000	−0.400	0.689	0.132	0.537	−1.820	0.069	0.128	0.239	−1.776	0.076
Evaluation	0.165	0.926	−0.796	0.426	0.163	0.894	−1.101	0.271	0.132	1.000	−0.034	0.973

** *p* < 0.001.

### Findings from think-aloud protocols

This section reports the findings from more fine-grained think-aloud protocols of two participants who received the process-genre writing instruction.

#### Jiaqi

Jiaqi was a freshman majoring in Computer Science. He had been studying an optional English course since he was in year three at primary school. After graduating from primary school, he entered a public secondary school, where English was a compulsory course. He did not receive systematic English writing instruction and had been exposed to only two genres of English writing (narrative and argumentative essay) at the time of the survey.

Analysis of Jiaqi’s think-aloud protocols produced in the pre-and post-test suggested that genre-based instruction significantly affected Jiaqi’s use of metacognitive strategies in planning, monitoring, and evaluating. His greater knowledge of the genre was also evident in these changes (Table 4).

**Table 4:** Jiaqi’s metacognitive strategy development trajectory.

Metacognitive Strategy use		Pre-test	Post-test
Planning	Time	limited	sufficient
	Content	random idea-generating	claims; evidence
	Organisation	Incomplete	argumentative essay structure
	Position	ambiguous	clear with audience awareness
Monitoring	Linguistic Level	grammar	vocabulary
		syntactic complexity	syntactic variety
	Discourse level	/	content; show evidence of using metalanguage
Evaluation	Time	limited	limited but more than pre-test
	Linguistic Level	grammar correctness	grammar; vocabulary; syntactic complexity
	Discourse Level	/	fluency; coherence
	Revision	surface level	surface level

Jiaqi’s metacognitive strategies for planning changed considerably in terms of the time spent on pre-task planning, the content and rhetorical structure for planning, and the writers’ positioning. In the pre-test, Jiaqi showed some awareness of planning but with limited effectiveness. He spent about 2 min and 54 s on pre-task planning. After reading the writing prompt, Jiaqi started to plan the content of his essay. However, his planning was brief and idea-generating, and the structure remained incomplete (Excerpt 1).

*Excerpt 1. Ok, now I start to read the prompts. The task states, [..] In the first paragraph, I should describe that with the development of society, online education is more and more prevalent. Then I will discuss the influence school education has on children, provide my opinion through contrastive analysis, and list the advantages and disadvantages of both school and online education to support my viewpoint. (Jiaqi, pre-test).*

*Excerpt 2. In the first paragraph, I should provide my thesis statement, which is that I disagree with the statement [..]. Then, in the second paragraph, I should claim that nowadays, teachers and students have a balanced relationship in teaching and practice. Then I will further elaborate on how this equality is beneficial to teaching. In the third paragraph, I would say teachers are respected and valued in modern society. Moreover, in the fourth paragraph, I first admit that in the past, teachers were respected and had very high social status, but now things are different from what they were in the past. I will support my claim by using a refutation. In the conclusion section, I will reiterate my opinion and emphasise that people’s appreciation of teachers and teachers’ values did not change, and teachers are even more valued in modern society. This is the outline of the essay. (Jiaqi, post-test).*

In the post-test, Jiaqi devoted more time to pre-task planning (7 min 30 s), in which his acquired genre knowledge was visible. He made explicit how he would organize the essay through which he showed a clear awareness of the rhetorical structure of argumentative writing (Excerpt 2). He demonstrated a marked improvement by constructing a defendable overarching claim, which shows his interpersonal metafunction awareness. The rhetorical structure planning indicates his understanding of textual metafunction. Following the structural planning, Jiaqi also offers his planning of ideational content for each paragraph, which suggests that he has some control of the ideational metafunction of the genre.

Jiaqi’s articulation of his metacognitive monitoring strategy in the pre-test and post-test showed that his monitoring strategy changed from the linguistic to the discourse level. In the pre-test, Jiaqi’s monitoring strategies were mainly concerned with grammatical accuracy and linguistic complexity (see Excerpts 3 and 4). Excerpt 3 shows Jiaqi’s perceived standard for a good quality essay: Using more complex sentences instead of the frequent use of simple sentences. Even though he was aware of using complicated syntactic structures, he did not indicate how these sophisticated sentences would help him convey ideas and achieve communicated purposes. He ended up using simple ones because he was afraid of making grammatical mistakes.

*Excerpt 3. “for example, people who”, again I used a subject clause. I can add more complex sentences to the essay rather than piling simple sentences. This can improve the quality of the essay. However, I cannot use complex sentence structure here because I am afraid of making grammatical mistakes.] (Jiaqi, pre-test)*

*Excerpt 4. But …, I used “although” in this sentence, so I cannot use “but” here (Jiaqi, pre-test)*

*Excerpt 5. This is the content for the first paragraph. I provided the background of the issue; that is, .., this is what I had written in the first paragraph (Jiaqi, post-test)*

*Excerpt 6. In the second paragraph, I feel it would be more reasonable to emphasise teachers' essential roles in modern society [..] That is the end of the second paragraph. (It) described teachers’ important role in the information age and elaborated on why they are still crucial in modern society. In the third paragraph, I will provide my second claim by explaining why teachers are valued and appreciated as they were in the past. (Jiaqi, post-test)*

Excerpts 5–6 display some of Jiaqi’s monitoring strategies in the post-test. It can be seen that his monitoring strategy moved from regulating linguistic accuracy and complexity to the appropriateness of content and writing progress. Excerpt 5 shows Jiaqi’s monitoring of the content for the first paragraph. After drafting the first paragraph, he did not continue with the second paragraph but reviewed what had been written in the first paragraph. In Excerpt 6, Jiaqi demonstrates his monitoring of the claims. He had initially planned to write his first claim, “teachers seem less appreciated in modern society because the way we express our respect and attitude has changed,” at the beginning of the second paragraph. Immediately after that, in this paragraph, he felt it would be more reasonable to elaborate on teachers’ essential roles in modern society. Evidence of such a monitoring process indicated that Jiaqi’s monitoring focus has shifted to a more discourse level than the surface level. Jiaqi read back what he had written in the second paragraph and planned subsequently what needed to be written in the third paragraph. Jiaqi’s backtracking to the previous text also showed his understanding of the recursive nature of the writing process.

Jiaqi’s awareness of evaluation was strengthened after the intervention. His increased articulation evidence this improvement in evaluating his essay and making corresponding revisions. In the pre-test, Jiaqi articulated three statements about evaluating, and all of them happened during the drafting process. Jiaqi’s evaluation focuses exclusively on surface-level textual features, with one account on vocabulary and two statements on grammar. Excerpt 7 shows Jiaqi’s evaluation of the complexity of the sentence. After coming up with the sentence “let me tell you some reasons,” he felt that this sentence was too simple and would affect the quality of the essay. He then expressed that he could not come up with any better sentence structures and would revise them later. This reveals Jiaqi’s weak awareness of argumentative writing style because he used an oral style sentence, “let me tell you.” It also shows his lack of lexical and syntactical resources. Although he wanted to improve the complexity of the sentence, he could not come up with any better ones. Excerpt 8 presents Jiaqi’s evaluation of grammatical accuracy. Although he realized the grammar was incorrect, he did not make a corresponding revision but continued drafting the essay. One possible reason for this might be that he was unsure how to revise the perceived grammatical problem; it may also be that the massive concurrent cognitive demand of generating ideas and constructing the sentence distracted his focus on grammatical revision.

*Excerpt 7. I intend to write “let me tell you some reasons”, but I think this sentence is too simple, which might affect the quality of the essay. But I cannot come up with a better one now, so I decided to keep it and move on. (Jiaqi, pre-test)*

*Excerpt 8. “There is no doubt the internet is already.. become..” I feel that the grammar is not correct. Em, the Internet has become the most popular tool. I intended to use the word tool, but I don't know how to spell it, so I used an alternative word, “way”, to express (the meaning of tool). (Jiaqi, pre-test)*

In the post-test, Jiaqi produced seven statements on revising. The most evident difference is that his evaluation and revising happened after drafting the whole essay, which is not observable in his pre-test transcripts. The after-draft revision shows changes in his writing strategies learned from the intervention. Jiaqi also devoted a longer time to revision than he did in the pre-test, suggesting that he possessed some features of proficient writers who spend substantial time reviewing and revision to meet their rhetorical goals (MacArthur et al., 2016). Although surface-level revision is still dominant in the post-test, it can be seen that Jiaqi also attempted to evaluate the content with the audience and purpose in his mind.

*Excerpt 9. Now I start to review (my essay). First, I think I did not meet the requirement of 300 words. Moreover, I think the syntactic structures are not varied. I used too many simple and limited complex sentences, and the vocabulary tended to be simple. (Jiaqi, post-test)*

*Excerpt 10. Here in the first paragraph, [..], I think I should use “for” instead of “to.” // Then in the second paragraph, “firstly, there is an information age, we need constant, we need constantly”, this should be an adverb, “constantly learning more than ever”//People always “place”, here I should notice the agreement issues. //This is the end of the review. There are several minor grammatical errors, and the flow of the essay is generally fluent. However, there are too many simple sentences, and using connectives is simple [..] That’s it. I think I should work on these areas in the future.] (Jiaqi, post-test).*

In Excerpt 9, Jiaqi demonstrates his use of revising strategy after he completed the draft. He evaluated the length, the syntactic variety and the lexical use of the essay. After that, he read the whole essay and made some surface corrections to grammatical errors such as the prepositions, agreement, tense, and adjectives & adverbs. He also pointed out his less frequent and simple use of connectives. Excerpt 10 shows Jiaqi’s revision process. He read through the essay and corrected some grammatical errors in prepositions, adjectives/adverbs, and agreement. This indicates that his revision attended to the surface-level linguistic features instead of the content or rhetorical structure. At the end of the review, he evaluated the essay as a whole. He was satisfied with the sentence construction by saying that “generally, the flow of the essay is fluent”, but he also realized he needed further improvement on vocabulary, sentence construction and more natural use of connectives. Jiaqi’s summary of the evaluation revealed that he attended to various aspects while evaluating the essay, such as grammar, fluency, coherence, syntactic variety and lexical diversity.

#### Chengji

Chengji was a freshman majoring in Economics. He has been studying English for 11 years, having started learning English in Grade 2 in primary school. Although English writing was not taught in primary school, he received some English writing instruction in secondary school, but the only genres were letters and argumentation. Think-aloud protocols show that he had strong metacognitive strategies, especially planning and monitoring, before the intervention. However, these strategies were used without incorporating genre knowledge. After the intervention, his knowledge of the argumentative genre was evident in his use of metacognitive strategies. Table 5 shows Chengji’s metacognitive strategy development trajectory.

**Table 5:** Chengji’s metacognitive strategy development trajectory.

Metacognitive Strategy use		Pre-test	Post-test
Planning	Time	sufficient	sufficient
	Content	both sides of the argument	claims; evidence
		general ideas	detailed supporting ideas
	Organisation	five paragraph but not genre specific	argumentative essay structure
	Position	clear	clear with audience awareness
Monitoring	Writing progress	length of text	length of text; time remaining
	Linguistic Level	grammar	language choice in considering the audience
		syntactic variety	
		lexical diversity	
	Discourse level	content	content; logic; coherence; rhetorical structures
Evaluation	Time	limited	limited
	Linguistic Level	grammar; spelling; word counting	grammar; lexical and syntactic variety
	Discourse Level	/	/
	Revision	surface level	surface level

The first significant change in Chengji’s planning strategy was a different focus when approaching the writing task. In the pre-test, after reading the writing prompt, Chengji identified the two sides of the argument, and immediately after, he attended to the time and the word limit for the task. Having a general perception of the writing topic and task requirement, he explicitly articulated that he would make a general plan for the essay. This overall task perception process suggested that Chengji was aware of pre-task planning and task requirements.

*Excerpt 11. After reading the task, we can see two sides to the argument. The viewpoint for one side is[..] The other side claims that[..]. Ok, I have 45 minutes for the task and need to write no less than 250 words. Now I am going to make an overall plan. (Chengji, pre-test)*

*Excerpt 12. Generally, I will look at this issue from both sides to discuss the argument dialectically. If I write opinions from both sides, it would be much easier to meet the requirement for the length. (Chengji, pre-test)*

In the post-test, however, Chengji’s attention was directed to the genre aspect of the task due to their awareness of the essay’s audience, the writer’s position and the identification of the genre.

*Excerpt 13. First of all, I suppose the audience of this essay should be teachers. Considering the essay’s audience, I think I should disagree with the argument [..]. (Chengji, post-test).*

*Excerpt 14. After reading the task, I think this is an argumentative task, and I plan to write a five-paragraph essay. The first paragraph introduces the background, and then I will provide my thesis statement, that is, [..]. In the second and third paragraphs, I will elaborate on my claims. My first claim is that teachers are important regarding their specific guidance.[..]. The second claim is that teachers’ role in developing students’ mentality cannot be ignored … In the fourth paragraph, I will refute the opposite idea.[…] The last paragraph will be the conclusion.[..]. This is the overall structure of this essay. Now I’m going to arrange the proportion for each paragraph. I should avoid writing too much at the beginning, and the major parts should focus on the development of the argument (Chengji, post-test).*

Excerpt 13 demonstrates Chengji’s awareness of considering the audience of the essay. Unlike his attention being focused on the word and time limit in the pre-test, Chengji, in the post-test, showed his awareness of considering the potential readers; this appeared to influence his position towards the argument and, importantly, identified the argumentative genre. Chengji’s change of focus on task perception from the pre-test to the post-test suggests that his planning shifted from planning the time and text length to considering the audience and defining the genre of the writing. Another notable change was observed in the planning of the organization. Chengji’s think-aloud transcript highlights the emergence of ideational content in organization planning after the process-genre instruction. In the pre-test, Chengji showed some awareness of planning the essay structure but did not identify the features of argumentative writing. In the post-test, Chengji planned the structure of the essay with a more nuanced understanding of the content, as shown in Excerpt 14. After reading the prompts, Chengji planned to write a five-paragraph argumentative essay. In the first paragraph, he was going to discuss the background of the argument, followed by the thesis statement. In the second and third paragraphs, he provided two claims with supporting evidence, using different methods such as stating facts and giving famous quotes. In planning the fourth paragraph, he also demonstrated knowledge about refutation, “Although the emerging of computers and technology might replace a teacher in some cases, the materials and online lessons are, in essence, designed by teachers.” In the fifth paragraph, he planned to conclude the essay by summarizing his opinions in the body paragraphs. After outlining the overall structure, he also showed an awareness of balancing each part of the article. He commented that the introduction should keep brief to avoid disproportion between the beginning and conclusion.

While Chengji demonstrated a high frequency of monitoring in both the pre-test and post-test, a close examination of the think-aloud transcripts revealed that, in the pre-test, his monitoring mainly concerned the writing progress (Excerpts 14 and 15) as well as vocabulary variety (Excerpt 16) and syntactic complexity (Excerpt 17). When Chengji realized that he had not used any complex sentences in his writing, he decided to use subordinate sentences to increase the “richness” of the essay. Chengji also considered sentence complexity a feature of a good article, and he purposefully attempted to achieve this goal. Throughout the writing process, Chengji tried to avoid repetition in the sentence structure.

*Excerpt 14. This is my first reason. 1.2.3.4… so far, I have written 14 lines, and there are about ten words in each line, so there are around 120 words in total. (Chengji, pre-test)*

*Excerpt 15. I am afraid I wrote too much for this paragraph. There is still half an hour; I need to hurry up. Anyway, I have written around 150 words. I need to hurry up. (Chengji, pre-test)*

*Excerpt 16. Because I have used “concentrate on” previously, here I need to change the expression to make the essay (vocabulary) more diversified. (I will use) “focus on”. (Chengji, pre-test)*

*Excerpt 17. Because I did not use attributive clauses in the previous text, nor did other subordinate clauses, I need to use more complex sentences to improve the essay's richness. (Chengji, pre-test)*

Chengji’s monitoring in the post-test suggested an enhanced understanding of the genre, which is evident in the use of metalanguage and focus on the logic and coherent development of the argument. Instead of attending too much to his essay’s progress and word counts, his focus was balanced on monitoring content, rhetorical structure, progress, sentence structures and lexical choices, as well as the logic and coherence of the essay.

*Excerpt 18. This is the beginning of the first paragraph. The background leads to my thesis statement. (Then) I can develop my argument (in the body paragraphs). The second paragraph mainly discusses the teacher’s role in guiding students. […] This is the content of the second (supporting) paragraph.[..] The fourth paragraph is a refutation. Although the Internet is developing rapidly and online education is becoming more popular, teachers ultimately obtain knowledge. (Chengji, post-test).*

*Excerpt 19. I think this sentence is illogical. There are some problems. So I will remove the phrase “only people value teachers can their children”] (Chengji, post-test)*

*Excerpt 20. I should use an attributive clause here, so it is a mixed-use of long and short sentences. After the attributive clause, I will add a short sentence, which can make the essay more coherent. (Chengji, post-test)*

*Excerpt 21. Because I used “value” too many times in my previous text, here I want to use “attach great importance to”; “they still attach great importance”. I try to pursue lexical diversity and variety in my essay so that it is more engaging to the readers. (Chengji, post-test).*

Excerpt 18 shows Chengji’s monitoring of writing progress and content. After finishing writing the first paragraph, he appeared confident about its content and purpose, that is, describing the background and providing a thesis statement. After reviewing the first paragraph, he discussed the second paragraph. Chengji also demonstrated his understanding of metalanguage, such as thesis statements and arguments. Similarly, Chengji monitored the content of the fourth paragraph and showed his knowledge about refuting the counterargument. Besides monitoring the writing progress and content, Chengji’s monitoring also involves careful consideration of the logic, as revealed in Excerpt 19. This indicates his understanding of logic as a critical feature of the argument genre. Excerpt 20 shows Chengji’s monitoring of the use of appropriate sentence structures. Chengji’s linguistic choices in the post-test served to develop a coherent essay, unlike his focus in the pre-test, which was on sentence variations to avoid repetition. Improvement in genre awareness was also observed in Chengji’s choice of lexical resources in the post-test. Instead of merely pursuing lexical diversity, he considered how the audience would be engaged in reading his essay (Excerpt 21)..

Regarding Chengji’s changes in evaluation, no evident change to his evaluation strategy after the process-genre instruction was found. Chengji’s evaluation strategy in the pre-test mainly focused on the length of the text and grammatical accuracy. Excerpt 22 relates that he counted the essay word by word to check whether he had met the task requirement after the drafting process. His focus was then on checking the spelling and grammar by reading his essay aloud to find possible spelling and grammar mistakes.

*Excerpt 22. Ok. Now I will check the total words. “1.2.3.4.5.6.7.8.9…” I think I meet the requirement of the task. Next, I will review the essay to see if there are any misspellings or grammatical errors.] (Chengji, pre-test)*

Compared to the evaluation in the pre-test, no evident changes were found in Chengji’s evaluation strategy in the post-test. Chegnji read through his drafted essay and found no grammar mistakes. He commented that his use of vocabulary is relatively simple, and syntactic variety needs further improvement. He then estimated the essay’s total words to ensure it met the task requirement. Unfortunately, Chengji did not comment on the content or organization of his writing, possibly because of the constraints of the test form and the time limit. As the think-aloud sessions used paper-based writing and were administered in a test environment, there was little opportunity to make substantive revisions to the content.

*Excerpt 23. This is what I have written for this essay. Now I will review the essay… (read the essay) … There are no obvious grammatical errors, and the vocabulary is relatively simple. Insufficient lexical resources lead to a simple expression. The syntactic variety needs improvement. The whole essay is around 300 words. (Chengji, post-test).*

## Discussion

Findings from the Metacognitive Strategy Questionnaire suggested that, while the process-genre instruction significantly improved participants’ consideration of the audience factor, no differences were found in the planning, monitoring and evaluation factor. However, the think-aloud protocols suggested that students’ metacognitive strategies in planning, monitoring and considering the audience were enhanced as the two participants purposefully incorporated their genre knowledge. Their genre-specific awareness of the audience, communicative purpose, language choices, style, and rhetorical structures were evident in their use of metacognitive strategies.

Participants in both groups showed no significant improvement in the planning factor of the MSQ, and there were no statistical differences between the two groups on the planning factors across the three tests. This may be due to the overconfidence of participants when reporting their metacognitive planning strategies in the pre-test, which leads to a relatively high mean score in the pre-test, leaving a slight margin for improvement in the post-test and delayed post-test. In the pre-test, most students indicated that they would plan the content, structure, language features, time and length of the essay according to their mean score of the planning factor. The analysis of the think-aloud protocols partly supports this assumption, as both participants performed some level of planning before writing in the pre-test. However, a detailed analysis of their protocols revealed that both participants were mainly concerned with random idea generation, or issues related to time or text length, without considering how to structure the essays with content knowledge relevant to the task. Such strategies are generally known as “knowledge telling” (Bereiter and Scardamalia, 1987) that are associated with novice and low proficiency writers (Manchón and Roca de Larios, 2007). They write down whatever topic-related ideas come into their mind without critically evaluating the appropriateness of the content in consideration of the writing purposes, audience and genre conventions (Harris et al., 2009). Similarly, Uzawa (1996) indicated that despite students having great metacognitive attention in the L1 and L2 writing, most of them generated ideas about what to say without considering strategies for organizing their ideas or arguing a point. These findings also echo Kessler (2021), who found that although two case study students self-reported many metacognitive awareness developments, one was unable to explain how she used her awareness for planning and evaluating and her evaluation was found to be superficial such as formatting evaluation. In the post-test think-aloud session, both students, who had received the process-genre instruction, spent more time on pre-task planning than they did in the pre-test. The planning was also more effective as they planned detailed content and rhetorical structures for their essay, indicating an awareness of the audience and communicative purpose. Deng et al. (2014) also found that, after the process-genre instruction, students planned appropriately using the overarching genre-based frames instead of writing directly after being given the topic. Manchón and Roca de Larios (2007) found that with increased proficiency, writers gradually devote more time to constructing pragmatic, textual and ideational representations of their writing in the planning process and incorporate them in their essay construction. This suggests that process-genre instruction, in this study, empowered the two participants to develop the planning strategies associated with more expert writers (Sasaki, 2000; Bai, 2018).

The MSQ showed that the process-genre instruction appeared to enhance participants’ consideration of the audience, while the conventional writing instruction had little effect. In the pre-test, participants’ mean scores for considering the audience factor suggested that they had only a moderate likelihood of considering the audience in the writing process. This finding corroborated what was found in the pre-test think-aloud, in which the two participants did not show awareness of considering the audience after they were given a writing prompt. This might be due to students’ experiences of writing on “context-free” topics as an assignment while having few opportunities to communicate with an authentic audience other than teachers. In the post-test questionnaire survey, a significant improvement was found in considering the audience factor by the experimental group, apparently as a result of explicit instruction in several stages of the process-genre instruction. The quantitative findings are also corroborated by the think-aloud transcripts, where one experiment student decided his position on the argument in considering his potential audience. Students’ enhanced audience awareness can be attributed to the process-genre intervention, where teachers activated students’ understanding of the three variables: audience, purpose and social context, reshaping students’ perception of the writing as a communicative tool in the “developing the context” stage. Likewise, Raphael et al. (1989) proposed that creating a communicative context enhances students’ awareness of the audience and purpose and their understanding of different aspects of the writing process. The peer review, during the reviewing stage, provides students with an alternative audience other than teachers. For the comparison group, the decontextualized instruction, or practice, may have constrained students from seeing writing as a social activity realized through the interaction of context, audience and communicative purpose. Many previous studies have acknowledged the impact of genre-based instructions on writers’ awareness of the audience and writing purposes (Myskow and Gordon, 2010; Yasuda, 2011; Negretti and McGrath, 2018). Our findings extend the understanding of how the Strategy-based process-genre approach enhanced writers’ audience awareness, which they apply in their writing process to achieve their communicative goals.

The monitoring factor in the MSQ elicits information on participants’ perceptions of having monitored their essay writing. Findings showed no significant differences in their perceived monitoring strategy use in the pre-, post-and delayed post-tests. This finding is in line with Zhang and Zhang (2022), who found that 8 weeks of teacher, peer or automated feedback had no significant differences in the effects of using self-monitoring strategies. One possible explanation could be due to the implicitness of monitoring instruction. Monitoring is a high executive ability with higher demands on working memories (De Silva and Graham, 2015; Bai, 2018). Unlike the planning strategy that can be shown with visual aids such as outlining and drawing mind maps, the demonstration of monitoring was implicit. Interestingly, analysis of the think-aloud protocols indicated that the two participants demonstrated a high level of monitoring during the writing processes both in the pre-test and post-test. A close examination of their think-aloud transcripts, however, indicated that the focus of the metacognitive monitoring strategy in the pre-and post-tests differed. In the pre-test, students were more likely to be concerned with monitoring the time remaining for the task and the spelling or grammatical correctness of the language, which is common in novice writers (Harris et al., 2009). This is primarily related to their exam-oriented learning experience, which directs their attention to those surface-level features. After the process-genre instruction, students in the experimental group focus on global and local monitoring with an increased genre awareness.

The evaluation factor assessed students perceived evaluating processes of language, organization, content and coherence. Metacognitive Strategy Questionnaire results indicated that students’ evaluating skills had not changed significantly at the post-test and delayed post-test for both groups. This finding is further supported in the analysis of the think-aloud protocols, with both participants demonstrating some awareness of evaluation in both pre-and post-tests. However, their evaluations were concerned only with checking grammar and task requirements. These findings suggested that the process-genre instruction did not develop learners’ awareness of, or ability to, evaluate their writing. Many researchers (e.g., Whalen and Ménard, 1995; Hayes, 2012; Graham et al., 2013) observed that when inexperienced writers were asked to revise, they attended primarily to local text problems, such as spelling and grammar, while ignoring text problems at the discourse level, such as organization and content; experienced revisers, in contrast, attended to problems both globally and locally. A possible reason for students’ lack of revision is that they do not know how to improve their vocabulary and sentence structures. This assumption supports Uzawa (1996), who found that even if some students identified errors when rereading their writing, they did not know how to revise them.

## Conclusion

This study examined the impact of the process-genre approach on writers’ use of metacognitive strategies in L2 writing. Findings show that the process-genre instruction significantly improved participants’ genre awareness, especially in the “considering the audience” factor, but no statistical differences were found in developing the planning, monitoring and evaluation aspect. The findings also revealed that conventional writing instruction had little effect on the comparison group’s use of metacognitive strategies in the four factors. Although statistical evidence supporting students’ enhanced planning, monitoring and evaluation strategies was not obtained, the results of the think-aloud protocols suggested that students used metacognitive strategies more effectively after the process-genre instructional intervention, especially in their pre-task planning and monitoring. Students have moved from surface-level linguistic features to more global ideational and textual-level planning and monitoring by incorporating their genre knowledge.

This study has some implications for L2 writing instruction. Teachers working with L2 students may ascertain valuable insights from the findings of this study on how the process-genre writing approach helped student writers develop their use of metacognitive strategies. Still, it is essential to note that not all strategies are developed congruently. Therefore, when using an integrated writing approach to teach a series of strategies, more attention to be paid to those less developed strategies such as evaluation. Besides modelling the evaluation and revising strategy used during the joint-construction stage, teachers are advised to design more substantial revising tasks to enhance students’ awareness of textual-level evaluation and revising. In designing writing tasks, a more explicit requirement on evaluation and revision can be included in the prompts so that students can realize the critical role of evaluation in the writing process. Moreover, we recommend that teachers understand students’ prior knowledge about strategy use before the instruction. When students demonstrate a high frequency of strategy use, it is essential to note whether their actual strategy use has incorporated genre knowledge and content knowledge to avoid students’ overconfidence in using strategies.

This study has some limitations due to the constraints of experimental conditions. For one, the results obtained from the quasi-experimental study should be interpreted with caution. This study is implemented in two intact College English classes with one experimental group and one comparison group. It would be meaningful for future studies to design two experimental groups with different proficiency levels to provide more insights into the effectiveness of the process-genre approach on students with different writing proficiency. The use of think-aloud protocols provided a deep understanding of students’ actual use of metacognitive strategies when engaging in writing tasks; however, due to the practicability and feasibility, we only examined the changes of two participants from the invention group. It is suggested that future studies employ some technology-based tools such as Keystroke Logging System (KLS) to track students’ writing and revision during the writing process (Bowen et al., 2022), with more participants involved to afford more robust findings about writers’ metacognitive regulation. Finally, our study only investigated students’ use of metacognitive strategies in the argumentative writing genre. Further studies are expected to examine the transferability of students’ metacognitive genre awareness across genres.

## Data availability statement

The original contributions presented in the study are included in the article/Supplementary material, further inquiries can be directed to the corresponding author.

## Ethics statement

The studies involving human participants were reviewed and approved by The University of Auckland. The patients/participants provided their written informed consent to participate in this study.

## Author contributions

YH conceived the initial idea, designed the study, collected and analyzed the data, and drafted the manuscript. LZ revised and proofread the manuscript. Both authors agreed to the final version before YH got it ready for submission as the corresponding author. All authors contributed to the article and approved the submitted version.

## Funding

This study is funded by Wuhan University Start-up Research Grants (No. 600460014).

## Conflict of interest

The authors declare that the research was conducted in the absence of any commercial or financial relationships that could be construed as a potential conflict of interest.

## Publisher’s note

All claims expressed in this article are solely those of the authors and do not necessarily represent those of their affiliated organizations, or those of the publisher, the editors and the reviewers. Any product that may be evaluated in this article, or claim that may be made by its manufacturer, is not guaranteed or endorsed by the publisher.

## Footnotes

^1^ https://www.cambridgeenglish.org/exams-and-tests/cefr/
