# Supplementary material for: Facilitating L2 writers’ metacognitive strategy use in argumentative writing using a process-genre approach
Source: Front Psychol. 2022 Nov 15;13:1036831. doi: 10.3389/fpsyg.2022.1036831 (PMC9707692; doi:10.3389/fpsyg.2022.1036831)
Supplement: Supplementary file 1 [file Data_Sheet_1.pdf]

Appendix A: Metacognitive Strategy Questionnaire

In this part, we would like you to help us by answering the following questions concerning your use of metacognitive strategies. Please give your answers sincerely, as only this will guarantee the success of the investigation. Thank you!

Please tell us how much you agree or disagree with the following statements by simply ticking (√) on the numbers from 1 to 7, following the instructions given below.

Name: ______________________

Gender: A. Male B. Female

Age: ______________________

Years of English learning: _______________

Not at all true of me	Not true of me	Slightly not true of me	Neutral	Slightly true of me	True of me	Very true of me
1	2	3	4	5	6	7

Before I start writing, I will...							
1. plan for the content of my essay.	1	2	3	4	5	6	7
2. plan how I am going to structure different parts of the essay.	1	2	3	4	5	6	7
3. plan what language features I am going to use in my essay.	1	2	3	4	5	6	7
4. I will think about how much time I should spend on the essay.	1	2	3	4	5	6	7
5. I will think about the length of my essay.	1	2	3	4	5	6	7
When I write, I try to ...							
6. think about what questions my reader might ask about the topic	1	2	3	4	5	6	7
7. think about my reader's views and opinions on the topic.	1	2	3	4	5	6	7
8. choose the level of formality of my essay to suit the reader.	1	2	3	4	5	6	7

Not at all true of me	Not true of me	Slightly not true of me	Neutral	Slightly true of me	True of me	Very true of me
1	2	3	4	5	6	7

When I am writing, I try to think about...							
9. how much time I have left	1	2	3	4	5	6	7
10. whether I am spelling some words correctly	1	2	3	4	5	6	7
11. whether I am using appropriate vocabulary.	1	2	3	4	5	6	7
12. whether I am using the correct grammar (e.g., tenses, prepositions, etc.)	1	2	3	4	5	6	7
13. how many arguments I should have in the essay	1	2	3	4	5	6	7
14. whether the argument follows the instruction of the essay	1	2	3	4	5	6	7
15. what parts my essay should have	1	2	3	4	5	6	7
16. how to connect different parts of my essay	1	2	3	4	5	6	7
After writing, I will...							
17. reread and evaluate my essay	1	2	3	4	5	6	7
18. make sure the language of my essay is clear	1	2	3	4	5	6	7
19. make sure the organisation is easy to follow.	1	2	3	4	5	6	7
20. make sure I have covered the content fully.	1	2	3	4	5	6	7
21. make sure all the paragraphs are relevant to the topic.	1	2	3	4	5	6	7
